# Endophytic Fungi as a Promising Source of Bioactive Compounds for Wound Healing: A Systematic Review

**DOI:** 10.3390/microorganisms14040918

**Published:** 2026-04-18

**Authors:** Marina Borges Guimarães, Carolina Castello Branco Rangel Helbourn, Gustavo Oliveira Gonçalves, Maria Beatriz Macedo Gonçalves, Damaris Silviera, Yris Maria Fonseca Bazzo, Paula Elaine Diniz do Reis, Pérola Oliveira Magalhães

**Affiliations:** Health Sciences School, University of Brasília, Brasília 70910-900, DF, Brazil; marina.borges@unb.br (M.B.G.); 251119230@aluno.unb.br (C.C.B.R.H.); 241129214@aluno.unb.br (G.O.G.); 251118439@aluno.unb.br (M.B.M.G.); damaris@unb.br (D.S.); yrisfonseca@unb.br (Y.M.F.B.); pauladiniz@unb.br (P.E.D.d.R.)

**Keywords:** tissue repair, cicatrization, secondary metabolites, natural products, in vivo and in vitro models

## Abstract

Endophytic fungi (EF) inhabit internal plant tissue in a mutually beneficial symbiotic relationship with their host plant. EF synthesizes metabolites that are structurally similar or identical to those found in their host plants, which include alkaloids, flavonoids, terpenoids, phenolic compounds, polysaccharides, proteins, lipids, and organic acids. These molecules have promising therapeutic effects, such as antimicrobial, antioxidant, anti-inflammatory, and antitumor activities. Wound healing has earned attention in recent years because of its relation to chronic pathological diseases. This systematic review scanned the available scientific literature database about the wound-healing properties of EF biomolecules. Amongst 994 works, 24 were screened after abstract and full-text reading. The studies were published between 2014 and 2026, in twelve countries. In total, 16 studies presented in vivo assays, 11 studies presented in vitro assays, and 3 studies presented both assays. Most studies identified molecules, which include melanin, benzoic acid, terpenes, sesquiterpenes (purpurolide), extracellular polysaccharides, exopolysaccharides, carotenoids, fatty acids, proteins, pyrones, quinones, and hydrocarbon acids, among others. A meta-analysis was not conducted due to high heterogeneity across extracts, methodologies, and outcomes. All studies showed wound-healing properties from EF extracts. The findings suggest a positive effect of EF extracts on wound-healing properties and the need for standardized in vitro and in vivo protocols.

## 1. Introduction

The skin is the largest organ in the human body with great adaptive capacity and plays several protective roles for the body, ranging from acting as a barrier against mechanical damage to preventing dehydration, as well as vitamin D synthesis, excretion, and absorption. In relation to wounds, the skin responds through the healing process, which can be divided into four main phases: hemostasis, inflammation, proliferation, and dermal remodeling [1]. First, hemostasis occurs to protect the vascular system and to form a cellular matrix that serves as a foundation for the subsequent phases of the healing process [2]. Next is the inflammatory phase, which acts as an innate body defense against potential pathogenic invasion caused by the wound [1]. This is followed by the proliferation phase, where processes such as re-epithelialization, angiogenesis, and fibroplasia occur. Finally, during dermal remodeling, the wound is repaired by skin that predominantly contains type I collagen [3].

Physical, chemical, thermal, microbial, or immunological damage can lead to skin wound formation [3,4]. The main causes of skin wounds include surgery, injuries, burns, or pathologic conditions (diabetes or vascular diseases) [5]. All wounds have the chance to become chronic, and some conditions can contribute to this conversion, such as venous or arterial insufficiency, diabetes mellitus, obesity, smoking, poor nutrition, infection, and immunological status [2,4,6]. Compounds with wound-healing properties can be obtained from natural products, including plants, animals, algae, fungi, bacteria, and lichens [7,8,9,10,11].

Fungi produce many primary and secondary metabolites—including terpenes, steroids, anthraquinones, and phenolic compounds—with documented antimicrobial activity against bacteria [4]. Endophytic fungi (EF) inhabit internal plant tissues and synthesize bioactive compounds that are structurally similar or identical to those found in their host plants. EF can be found in all plant parts and tissues, including the roots, stems, leaves, fruits, flowers, bark, and scales [12]. A variety of EF species can be isolated from one single host plant, which is related to the intrinsic characteristics of the host plants, such as the isolation tissue, geographic location, growth period, collecting season, and their variety [13]. As a consequence of their diversity, a range of metabolites, which include alkaloids, flavonoids, terpenoids, phenolic compounds, polysaccharides, lipids, organic acids, proteins, and others, have been isolated from EF. These metabolites are reported to have promising pharmacological effects such as antimicrobial, antioxidant, and anticancer properties. The structural diversity of these compounds represents a valuable resource for natural product discovery [14].

Endophytic fungi have emerged as promising sources of bioactive compounds with potential applications in wound healing. Studies have shown that EF isolated from *Garcinia* species exhibits significant antimicrobial activity against common wound-associated pathogens such as *Escherichia coli*, *Staphylococcus aureus*, *Saccharomyces cerevisiae*, *Geotrichum* sp., and *Penicillium canadensis* [15], suggesting their potential to prevent or control wound infections. A *Fusarium tricinctum* isolate showed notable free radical scavenging activity, comparable to that of vitamin C [16], showing its role in mitigating oxidative stress, a key factor in delayed wound healing. Several secondary metabolites produced by EF, such as polyketides and alkaloids, have been reported to possess anticancer and regenerative properties [17]. Wound healing activity is reported to be related to aromatic acids, phenolic compounds, anthraquinones, terpenoids, glycosides, polysaccharides, alkaloids, peptides, polyketides, and saponins [18]. However, there are a few reports in the literature focusing on wound-healing properties.

In this context, this study aimed to conduct a systematic review to assess the evidence that EF is a source of biomolecules that promote wound healing. Given the growing interest in natural sources, especially in the healthcare field, this review sought to compile and analyze a few studies published on the topic, considering both in vivo and in vitro research, thus providing a foundation for future studies. In addition, this review brings updates, up to 2026, focusing only on endophytes isolated from plants. Included papers were selected with strict eligibility criteria, and they were individually evaluated, especially regarding their methodologies and main findings. In this scenario, an updated systematic review will be essential for understanding the role of endophytic fungus metabolites in wound healing, as well as the delivery systems involved.

## 2. Materials and Methods

This review was conducted using the Preferred Reporting Items for Systematic Reviews (PRISMA 2020, [19]). The systematic review protocol was registered in PROSPERO (Registration No.: CRD420251054403 [20]).

### 2.1. Eligibility Criteria

The review question “Are Endophytic Fungi A Source Of Biomolecules With Wound Healing Activity?” was built using the PICOS acronym, where P is for “cells and/or animals with inducted wounds”, I is for “extracts and/or compounds from endophytic fungi”, C is “the substrate that does not contain the endophytic fungi extract and/or compound (the vehicle)”, O is for “wound healing”, and S is for “experimental studies (randomized and non-randomized)”. The inclusion and exclusion criteria were defined according to the review question and are described below.

### 2.2. Inclusion Criteria

We included studies that focused on wound healing activity, in both in vivo and in vitro assays, of biomolecules from endophytic fungi. The biomolecules can be present in a raw extract, in a vehicle, or as an isolated compound.

### 2.3. Information Sources and Search Strategy

Detailed individual search strategies for each of the following bibliographic databases were developed: PMC via National Library of Medicine (NLM), PubMed via NLM, EMBASE via Elsevier, Web of Science Core Collection (WoSCC) via Clarivate, Scopus via Clarivate, and Science Direct. Gray literature was also assessed from Google Scholar and the ProQuest^TM^ Dissertation & Theses Citation Index via Clarivate, as well as hand searches of bibliographies from included studies (Table S1 (Supplementary Material)). The search included all studies published until 10 March 2026 across all databases without language restriction. In addition, the reference lists of the selected articles were carefully checked for potentially relevant studies that could have been overlooked during the electronic database search, and experts in the field were consulted. Duplicated references were removed using the EndNote^TM^ (version 21.5) reference manager software (Clarivate Analytics) [21].

### 2.4. Exclusion Criteria

The exclusion criteria were (1) observational studies, reviews, conference abstracts, editorials and expert opinion; (2) inducted wounds in humans; (3) inducted wounds in plant tissue; (4) cells and/or animals without inducted wounds; (5) extracts and/or compounds from filamentous fungi, bacteria, endophytes, and any other organism rather than endophytic fungi; (6) compounds from non-identified endophytic fungi species; (7) synthesized molecules; (8) wound-healing assays in cancer cells; (9) fungi isolated from other organisms besides plants; and (10) full text not found even after contacting authors. All criteria are listed in Table S2 (Supplementary Material).

### 2.5. Study Selection

Study selection was completed in two phases. In the first phase, two reviewers (CCBH and GOG) independently reviewed the titles and abstracts of all references found from the database search, using EndNote^TM^. The reviewers selected studies based on their titles and abstracts that matched the inclusion criteria. A third reviewer (MBG) was consulted when disagreements emerged between the two initial evaluators. In the second phase, two reviewers (CCBH and MBMG) read full-text studies, and the ones that did not fulfill the inclusion criteria were excluded. In case of any disagreements, MBG was consulted.

### 2.6. Data Collection Process and Data Items

The whole process—from the isolation of endophytic fungi from plants to the cultivation of endophytic fungi, the extraction of bioactive compounds from endophytic fungi, the characterization of bioactive compounds, and the bioactive evaluation of the compounds—is illustrated in Figure S1 (Supplementary Material).

Two reviewers collected the information required from the selected articles. All full-text articles were independently reviewed. Any disagreement was resolved by mutual agreement between reviewers. The following information was recorded for all included studies: author(s), year, country of publication, plant species, fungus species, growth conditions (medium composition, temperature, agitation speed, and time), extraction method, biomolecule, wound-healing assay (cell type), in vivo assays, and other complementary assays (antimicrobial activity, anti-inflammatory, antioxidant activity, etc.).

### 2.7. Risk of Bias in Individual Studies

No specific quality assessment method was developed for the in vitro studies included in this review. The method of the selected in vitro studies was evaluated using the scientific criteria proposed by Greenhalgh [22] and adapted according to Wanderley et al. [23]. CCBH and GOG scored each item as ‘high’-, ‘moderate’-, or ‘low’-quality and independently assessed the quality of each included in vitro study. Any disagreements were resolved by consulting MBG. *Fungus source and isolation*: description of the species used, details of the host plant (species, part used, geographic location, and collection data), description of the fermentation conditions used to cultivate the fungus. *Extraction conditions*: extraction methods describing the amount of biomass/supernatant used, the solvent and the amount of solvent, and the final product used in assays. *Wound-healing assay*: cell line, the purpose of the study, dose, timing, and use of controls. *Mechanism of action*: assays for the elucidation of the mechanism of action of the molecule and discussion about the mechanism of action.

The Collaborative Approach to Meta-Analysis and Review of Animal Data from Experimental Studies (CAMARADES) checklist was used to assess the quality of in vivo studies [24]. The tool contained 10 questions that assessed whether there was a bias in the study design. With the highest score of 10, a higher score assessed for the study showed a better methodological quality of the study [25]. Each included in vivo study was analyzed according to the CAMARADES checklist by all the reviewers. Disagreements were resolved through discussion and mutual agreement among the three reviewers.

### 2.8. Declaration of Generative AI in Scientific Writing

During the preparation of this study, ChatGPT(OpenAI, GPT-5.3 version) was used to improve the readability and language of the manuscript and for the translation of non-English or Portuguese studies. After using this tool/service, the authors reviewed and edited the content as required and take full responsibility for the publication.

## 3. Results

### 3.1. Study Selection

**Figure 1:**
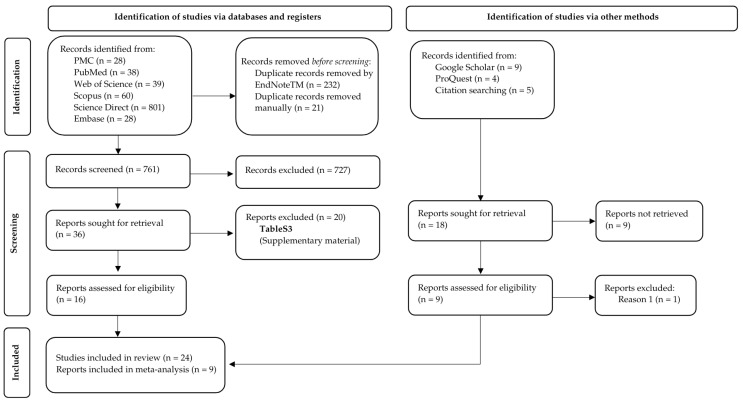
Diagram of literature search and selection criteria. Adapted from Page et al. [19].

In this work, 994 citations were identified from six databases: PMC, PubMed, Web of Science, Scopus, Embase, and Science Direct. A total of 232 duplicates were detected by EndNote^TM^, and 21 additional duplicates were removed manually. After their removal, 761 citations remained. A comprehensive evaluation of the titles and abstracts was completed, and 36 research papers remained after this phase. All studies were submitted to a full-text review by the reviewers, and this process led to the exclusion of 20 papers, leaving 16 remaining (Figure 1). Through gray literature screening (Google Scholar, ProQuest, and hand searches of bibliographies from included studies), 18 studies were identified, 8 were assessed for eligibility, and, finally, 8 were included in the review. As a result, 24 studies were included in the review. The exclusion reasons for each study are reported in Table S3 (Supplementary Material).

### 3.2. Study Characteristics

The included studies were conducted in twelve countries—Brazil [26], China [27,28,29,30], Egypt [31,32,33,34,35,36], India [37,38], Indonesia [39], Iran [40], Malaysia [41,42], Mexico [43], Saudi Arabia [44], South Korea [45,46,47], Tanzania [48], and Uzbekistan [49]—published between 2014 and 2026, all published in English except for Araújo [26] (Portuguese) and Abdulmyanova et al. [49] (Russian) (Table 1). All selected studies addressed wound healing activity from EF extracts, of which 16 presented in vivo assays. Most in vivo studies used mouse models, but also studies with rat, rabbit, and earthworm models were reported.

The selected studies reported EF isolated from plants. The majority described how isolation was conducted, including which methods included rinsing/washing plant parts with ethanol (70%, 75%, 95%, and 100%), NaClO (2% and 4%), and distilled water [26,28,32,38,48,50]; with ethanol (70%) and distilled water [33,35,51]; or with ethanol (70% and 75%), HgCl_2_, and distilled water [34,36]. The remaining studies did not report how the isolation from plants was executed, and only claimed that the fungus came from a plant part [27,29,31,37,39,40,41,42,43,45,46,47,49].

**Table 1:** Summary of results from the selected studies.

Country	Plant Host	Fungus	Extraction Method	Wound- Healing Assay	Molecule	In Vivo Model	Main Conclusions	Author, Year
Uzbekistan	*Helianthus* *tuberosus*	*Cladosporium* sp.—HT207	Mycelium alkaline extraction + acid precipitation	In vivo assay with ointment	Melanin	Rabbit	A 5% melanin-based ointment from *Cladosporium* sp.—HT207 presented wound-healing properties in stomatitis and a protective effect against UV radiation.	Abdulmyanova et al. (2023) [49]
Saudi Arabia	*Reseda* *arabica* (leaves)	*Aspergillus* *parasiticus*	LLE with EtOAc	In vivo assay with AgNPs	ND	Mice	The AP-AgNPs appeared to promote wound healing, in addition to exhibiting promising antibacterial activity for the treatment or prevention of infections caused by methicillin-resistant *Staphylococcus aureus*.	Ali et al. (2024) [44]
Malaysia	Sea weed *(Gracilaria arcuata Zanardini, Gracilaria coronopifolia J.Agard* and *Acantophora spicifera)*	Three marine endophytic fungi (CN, NM and ZD)	LLE with EtOAc	In vivo assay with extract in Tween 20^®^ + distilled water as vehicle	ND	Sprague-Dawley rats	The study demonstrated the wound healing activity of three marine endophytic fungi extracts on second-degree burn wound healing. The extracts were found to be effective as compared to the commercial wound healing drug, sulphadiazine, in Sprague-Dawley rat models.	Aqilah et al. (2018) [42]
Mexico	ND	*Daldinia* *eschscholtzii* (Ehrenb.) Rehm	LLE with EtOH	In vivo assay with ointment	ND	Mice	The ointment promotes effective and safe healing in mice.	Cueva-Clavijo et al. (2024) [43]
Brazil	*Acrocomia aculeata* and *Poincianella pyramidali*s	*Penicilium* sp. and *Rhizoctonia* sp.	LLE with EtOAc	In vitro assay with 3T3 cells	ND	ND	The fungal extracts showed similar and positive wound healing activities, with the *Penicillium* sp. extract being slightly superior, making the result promising. Moreover, this same extract was the only one that showed activity against *Staphylococcus* strains.	Araújo (2018) [26]
Egypt	ND	*Aureobasidium pullulans* AKW	Acid extraction and cold precipitation	In vitro scratch assay with skin fibroblast cell	Melanin	ND	The melanin extracted from *Aureobasidium pullulans* AKW exhibited significant wound healing activity, in addition to antioxidant properties in human cells.	Elattar et al. (2024) [32]
Egypt	*Cucumis sativus* (cucumber) leaves	*Aspergillus niger*	SLE with EtOAc	In vitro assay with human fibroblast cells W138 and vivo assay	Pyrones and quinones	Wistar albino rat	The EtOAc extract revealed 15 compounds (pyrones and quinones, mainly), which presented antimicrobial activity against *S. aureus* and biofilm reduction. The extract promoted wound healing and anti-inflammatory properties in human fibroblasts.	El-Bouseary et al. (2025) [33]
Egypt	*Cucumis sativus* (cucumber) leaves	*Rhodotorula* *mucilaginosa*	SLE with EtOAc	In vitro assay with W138 cells	Carotenoids and fatty acids	Wistar albino rats	The EtOAc extract provided in vitro and in vivo antimicrobial, anti-inflammatory, and wound-healing properties.	Eliwa et al. (2025) [35]
Egypt	*Hibiscus rose-sinensis* (leaf) *Azadirachta indica* (twig) *Ricinus communis* (twigs) *Ricinus* *communis* (leaves)	*Fusarium equiseti* *Aspergillus terreus* *Aspergillus* *quadrilineatus* *Aspergillus ochraceus*	Addition of sodium selenite to the culture media	In vitro assay with human skin fibroblast cell lines	Fatty acids, hydrocarbon compounds, and proteins	ND	Growth conditions were optimized for a higher yield of EF strain SeNPs, which offered potential anticancer and wound healing activities.	El-Sayed et al. (2026) [34]
Egypt	*Lycium shawii*	*Neurospora crassa* SSN01	LLE with EtOAc, MetOH and Hex	In vivo assay (BA-based ointment)	Benzoic acid (BA)	Rabbit	BA aided healing and prevented the adverse effects of silver sulfadiazine	El-Zawawy et al. (2022) [36]
Egypt	*Cucumis sativus* L. (leaves)	*Penicillium* *rubens*	LLE with EtOAc	In vitro assay with WI38 cells	ND	ND	The treatment with the fungal extract increased cell migration in the wounds and significantly promoted wound closure compared to the control. Additionally, it showed a significant anti-Pseudomonas effect.	Farghali et al. (2025) [51]
Iran	*Olea europae* L. (Olives)	*Penicillium* *terrestris* PT22AV	Precipitation with cold EtOH	In vivo assay with EPS solutions	EPS of 202 kDa	Wistar rats	EPS from *P. terrestris* presented wound healing potential and antioxidant and antibacterial properties.	Hamidi et al. (2023) [40]
South Korea	*Pinus* *densiflora* (leaves)	*Talaromyces* *purpureogenus* (MK108915)	SLE with H20	In vitro wound scratching assay on NIH3T3 cells	ND	ND	Tp-AgNPs exhibited significant wound healing activity, were non-toxic to the tested cell line, and showed good inhibitory effects against various pathogenic bacteria.	Hu et al. (2019) [46]
South Korea	*Pinus* *densiflora* (leaves)	*Talaromyces* *purpureogenus* (MK108915)	Precipitation with EtOH	In vitro assay on HEK293 cells	Extracellular polysaccharides	ND	The polysaccharides TEPS1 and TEPS2 exhibited wound healing activity; however, TEPS1 showed a higher wound healing activity than TEPS2, in addition to exhibiting a promising antioxidant activity.	Hu et al. (2023) [45]
China	*Caesalpinia* *sepiaria* (leaves)	*Diaporthe* *unshiuensis* (YSP3)	LLE with EtOAc	In vivo assay with *Diaporthe unshiuensis* carbon dots (Du-CDs)	ND	Mice	Du-CDs featured an improved antimicrobial effect against both bacteria and fungi in comparison with the fungal extraction, and also accelerated wound healing ability, with satisfactory results in terms of in vivo biocompatibility.	Khan et al. (2024) [27]
China	*Edgeworthia chrysantha* (leaf tissue)	*Penicillium* *purpurogenum*	LLE with EtOAc and ultrasound	In vivo assay with raw extract	Purpurolide	Mice	Purpurolide C (PC) from *P. purpurogenum,* formulated for transdermal use, was reported to show diabetic wound healing-promotion effects by inhibiting inflammatory macrophage activation.	Liu et al., (2023) [28]
India	*Aegiceras* *corniculatum*	*Arthrinium* *aureum*	M.FeONPs synthesized from fungal supernatant	In vitro (HUVEC cells), and in vivo	ND	Mice	The MFeONPs are distinguished from iron-synthesized nanoparticles. MFeONPs exhibited lower toxicity, superior pro-angiogenic properties, and enhanced wound healing activity.	Mandarada et al. (2025) [37]
Tanzania	*Jatropha* *multifida* (leaves)	*Phlebiopsis* *gigantea,* *Phyllosticta* sp., *Colletotrichum* sp., and *Phyllosticta* *elongata*	LLE with EtOAc	In vivo assay with raw extract	Alkaloids, flavonoids, phenolics, saponins, and tannins	Mice	Crude extracts from endophytic fungi, especially *P. gigantea* (FUCE 1), significantly accelerated wound contraction in mice and reduced clotting time. FUCE 1 had high levels of bioactive compounds.	Mpenda et al. (2024) [48]
South Korea	*Quercus* *rubera* L.	*Penicillium* *radiatolobatum*	LLE with EtOH	In vitro assay with NIH3T3 cells	ND	ND	The GA-CU-CeO_2_ NCs exhibited significant wound healing activities and good antioxidant properties.	Naveen et al. (2024) [47]
India	*Xylaria* *arbuscula*	*Blumea axillaris*	SLE with water	In vitro assay with L929 cells	ZnONPs	ND	The *B. axillaris* ZnONPs promoted healing activity in a dose-dependent manner	Nehru et al. (2023) [38]
Egypt	*Cornulaca* *monacantha* (stem samples)	*Paecilomyces* sp. (AUMC 15510)	LLE with EtOAc	In vivo assay with raw extract	Phenolic compounds and flavonoids	Earthworm	The EtOAc extract from *Paecilomyces* sp. (PsEAE) exhibited antimicrobial activity and antibiofilm and wound-healing properties. In vivo models treated with Vaseline with PsEAE presented a faster healing process than models with only Vaseline.	Salem et al. (2022) [31]
Indonesia	*Dahlia variabilis*	*Aspergillus* *fumigatus*	ND	In vivo assay with endophytic fungal extract	Mainly terpenoids	Mice	*A. fumigatus* extracts (at 5%) promoted collagen regeneration and mitigated inflammation in Candida albicans-infected wounds.	Shinta et al. (2024) [39]
Malaysia	*Orthosiphon stamineus*	*Penicillium* *minioluteum* ED24	SLE with CH_2_Cl_2_ followed by chromatography with Hex, EtOAc, and MetOH	In vivo assay with the MaB10 fraction (MetOH)	ND	Sprague-Dawley rats	The wounds treated with the Ma10 fraction samples in petroleum jelly showed significant wound healing activity, especially at the 2% concentration.	Yenn et al. (2014) [41]
China	*Orchidantha chinensis*	*Penicillium* *spinulosum* OC-11	SLE with H2O, followed by incubation with AgNO_3_	In vivo assay with AgNPs	ND	Sprague-Dawley rats	The proteins produced by *P. spinusolum were* capped on the AgNPs and secured the nanoparticles with low aggregation. AgNPs presented antimicrobial and wound healing activities.	Wen et al. (2016) [29]

AgNPs: silver nanoparticles; BA: benzoic acid; CH_2_Cl_2_: dichloromethane; EPS: exopolysaccharide; EtOAc: ethyl acetate; EtOH: ethanol; GA-CU-CeO_2_ NCs: gum arabic–curcumin–CeO_2_ nanocomposites; LLE: liquid–liquid extraction; M.FeONPs: iron-oxide nanoparticles; MetOH: methanol; ND: non-declared; SeNPs: selenium nanoparticles; SLE: solid–liquid extraction.

### 3.3. Host Plants and Fungus Species

Several plant species were cited as hosts of EFs throughout the study, with no observed prevalence regarding species, genus, or family (Table 1).

The analyzed studies covered a diversity of plant hosts, including *Edgeworthia chrysantha* [28], *Orchidantha chinensis* [29], *Cornulaca monacantha* [31], *Jatropha multifida* [48], *Caesalpinia sepiaria* [27], *Orthosiphon stamineus* [41], *Reseda arabica* [44], *Pinus densiflora* [45,46], two undeclared species treated in separate studies [32,45], three sea weed species [42], *Lycium shawii* [36], *Quercus rubera* L. [47], *Helianthus tuberosus* [49], *Olea europae* L. [40], *Dahlia variabilis* [39], *Aegiceras corniculatum* [37], *Cucumis sativus* L. [33,35,51], *Xylaria arbuscula* [38], *Acrocomia aculeata* and *Poincianella pyramidalis* [26], *Hibiscus rose-sinensis*, *Azadirachta indica*, and *Ricinus communis* [34]. Among the studies, nine used leaf samples: Ali et al. [44], El-Bouseary et al. [33], Eliwa et al. [35], El-Sayed et al. [34], Farghali et al. [51], Hu et al. [45,46], Khan et al. [27], and Mpenda et al. [48]. Liu et al. [28] studied the use of leaf tissues; Salem et al. [31] reported stem samples [31]; Hamidi et al. [40] used olives; and El-Sayed et al. [34] used not only leaves, but also twigs. The remaining studies did not specify in which part of the plant the EFs were isolated.

Different fungal genera were addressed in the studies; however, there was a higher frequency of representatives of the genus *Penicillium*, reported in 30% of the studies [26,28,29,40,41,47,51], followed by *Aspergillus* [33,34,39,44] (16%), and *Talaromyces* [45,46], present in 8% of the publications (Table 1). The remaining endophytic fungal genera accounted in the selected articles include *Cladosporium*, *Daldinia*, *Rizhoctonia*, *Fusarium*, *Neurospora*, *Aureobasidium*, *Rhodotorula, Diaporthe*, *Arthrinium*, *Phlebiopsi*, *Colletotrichum*, *Phyllosticta*, *Blumea*, and *Paecilomyces* [26,27,31,32,35,36,37,38,40,42,43,48,49]. Only one study did not identify the EF species [42].

### 3.4. Growth Conditions and Extraction Methods

The observed cultivation conditions revealed that the most used culture medium for the growth of EF was Potato Dextrose (PD) and its variations, in solid form (PDA) or liquid (PDB), present in 63% of the studies [26,27,31,32,34,36,37,39,42,44,45,46,47,48,49]. Other culture media accounted for 37% of the studies [28,29,33,35,38,40,41,43,51]. Overall, 55% of the studies used cultivation under agitation [27,29,31,32,38,39,41,44,45,46,48,49], while 20% used stationary conditions [33,34,35,36,42]. In 25% of the articles, agitation conditions were not declared [26,28,37,43,47,51]. Fungi were cultivated at an average temperature of approximately 25 °C, with few variations among the studies.

Regarding the extraction method for bioactive metabolites, liquid–liquid extraction (LLE) was the most employed, being used in 55% of the studies [26,27,28,31,33,35,36,42,43,44,47,48,51]. Solid–liquid extraction (SLE) was reported in 20% [29,38,41,46,49], while 20% used different extraction methods [32,34,37,40,45]. Finally, one study specified neither the method nor the solvent used for obtaining the extract [39].

### 3.5. Bioactive Compound Characterization

The characterization of molecules described in the selected studies was performed by various methods, including chromatographic, spectroscopic, and microscopic techniques. Chromatographic methods such as gas chromatography–mass spectrometry (GC-MS), high-performance liquid chromatography (HPLC), and liquid chromatography–mass spectrometry (LC-MS) were used. In addition, Fourier-transform infrared spectroscopy (FTIR) and nuclear magnetic resonance (NMR) were employed as spectroscopic methods. To evaluate morphological properties, scanning electron microscopy (SEM) and transmission electron microscopy (TEM) were used.

Among the 24 included studies, eight did not report the molecule used [26,27,29,37,42,44,46,51]; on the other hand, three studies described the use of nanoparticle-based delivery systems [34,38,47]. Abdulmyanova et al. [34] reported the biomolecule melanin; however, no detailed information regarding its identification or characterization methods was provided. Cueva-Clavijo et al. [43] identified a complex mixture of compounds using gas chromatography–mass spectrometry (GC-MS).

El-Zawawy et al. [36] did the identification of benzoic acid using multiple analytical techniques, including thin-layer chromatography (TLC), UV-Vis spectrophotometry, Fourier-transform infrared spectroscopy (FTIR), GC-MS, and high-performance liquid chromatography (HPLC). Similarly, Elattar et al. [32] characterized melanin using FTIR, 1H-NMR spectroscopy, mass spectrometry, and scanning electron microscopy (SEM). Hu et al. [45] characterized exopolysaccharides using HPLC, FTIR, and NMR, whereas Hamidi et al. [40] employed FTIR, SEM, size-exclusion HPLC, X-ray diffraction, and GC-MS for exopolysaccharide characterization. Liu et al. [28] used dynamic light scattering (DLS) and transmission electron microscopy (TEM) to characterize porpurolide C, a sesquiterpene.

In the study by Mpenda et al. [48], compound identification was limited to the detection of metabolite classes (alkaloids, tannins, flavonoids, phenolics, and saponins) through qualitative phytochemical screening based on colorimetric and precipitation tests. Salem et al. [52] characterized flavonoids and phenolic compounds using HPLC and GC-MS, while Shinta et al. [39] identified terpenoids via GC-MS. El-Bouseary et al. [33] analyzed pyrones and quinones using liquid chromatography–mass spectrometry (LC-MS), and Eliwa et al. [35] identified carotenoids and fatty acids through high-resolution LC-MS (HR-LCMS).

### 3.6. Wound-Healing Assay

#### 3.6.1. In Vitro Studies

In this systematic review, eleven studies determined wound-healing properties through in vitro methods, including the wound scratch assay. This assay comprises creating a scratch with a sterile pipette tip in the cell monolayer. The cell types varied, but 82% of the studies used fibroblasts, including WI-38, 3T3, NIH3T3, BJ1, and L929 cell lines [26,32,33,34,35,38,46,47,51]. Only 18% of the studies did differently. Hu et al. [45] used immortalized human embryonic kidney cells, HEK293, and Mandarada et al. [37] used HUVECs, a type of cell derived from the endothelium of human umbilical cord veins (Table 2). Moreover, the negative control of all studies was untreated cells, and only Mandarada et al. [37] used a positive control, vascular endothelial growth factor.

To calculate the wound closure, they used the formula below:

$$Wound\;closure\left(\%\right)=\frac{\left(A_{0}-A_{t}\right)}{A_{0}}\times 100$$

in which A_0_ is the initial wound area, and A_t_ is the wound area after a pre-established time. All studies calculated the wound areas using ImageJ software(version 1.53).

All the studies exhibited positive results in wound closure in treated groups, compared to controls. In total, 75% of the studies showed wound closure with the treatments at 24 h; however, Naveen et al. [47] showed that the control group also experience closed wounds. Hu et al. [45] only documented at 36 h, and Mandarada et al. [37] only documented at 8 h, and suggested that the wound closure was higher after that.

In the study by De Araújo [26], the effect of ethyl acetate extracts of *Penicillium* sp., *Rhizoctonia* sp., and an unidentified endophytic fungus on 3T3 fibroblast migration was evaluated. The study found that after 24 h, the results of ethyl acetate extracts of *Penicillium* sp. and *Rhizoctonia* sp. were like the control (44.81 ± 4.89%), being 44.18 ± 3.47% and 46.98 ± 5.05%, respectively. However, the unidentified fungus showed a higher result than the control of 49.94 ± 4.03%.

Farghali et al. [51], El-Bouseary et al. [33], and Eliwa et al. [35] tested raw EF extracts against WI31 cells. Farghali et al. [51] tested extracts from the endophytic fungus *Penicillium rubens* (EPR). Cell migration into the wound area was visualized using phase-contrast microscopy. The relative wound area was evaluated at 0, 24, and 48 h post-scratch. As a result, the treatment with EPR significantly increased the wound closure percentage (66.64% ± 5.61) compared to the control cells (13.79 ± 3.98%) at 24 h post-wound induction. Also, the wound closure percentage significantly increased in EPR-treated cells (99.94± 0.05%) compared to the control ones (83.37 ± 0.05%) at 48 h. Both El-Bouseary et al. [33] and Eliwa et al. [35] tested ethyl acetate extracts, referred to as ANM (from *Aspergillus niger*) and ERM (from *Rhodotorula mucilaginosa*), respectively. Treatment with ANM resulted in 52.37 ± 2.4% of wound closure after 24 h, whereas the control group showed 13.79 ± 3.98%, which was significantly lower. Similarly, ERM treatment increased wound closure to 42.68 ± 3.43% compared to the control group (13.79 ± 3.98%).

Both Naveen et al. [47] and Hu et al. [46] did the in vitro wound healing with NIH3T3 cells. In Naveen et al.’s [47] study, NIH3T3 cells with scratches were tested with CeO_2_ nanoparticles and gum arabic–curcumin nanocomposites (GA-CU-CeO_2_ NCs from *Penicillium radiatolobatum*), and wound healing was monitored over 0–36 h using light microscopy. Hu et al. [46] evaluated the Tp-AgNPs’ (*Talaromyces purpureogenus* silver nanoparticles) wound healing effect over 0–48 h using light microscopy. Results of Naveen et al. [47] demonstrated that GA-CU-CeO_2_ NCs presented excellent proliferative and migratory capabilities over different incubation times. There was increased proliferation and migration of NIH3T3 cells at 12 h, and at 24 h, the wound closure was improved in all tested groups, including the control. Hu et al. [46] reported the closure of the wound area with the treatment with Tp-AgNPs compared to the untreated group after 24 h, in a dose-dependent manner. The wound area was 2.88, 3.42, and 2.54 cm^2^ at different concentrations (1, 5, and 10 μg/mL, respectively), while the negative control was 3.79 cm^2^.

Hu et al. [45] cultured scratched HEK293 cells with two extracellular polysaccharides from *T. purpureogenus* endophytic fungus (TEPS1 and TEPS2), and evaluated the wound closure for 0—36 h. Results showed that, after 36 h, TPS1 promoted complete wound closure, while the control group had an unrecovered wound area of 5.37 cm^2^, and TEPS2 had an area of 4.48 cm^2^. Nehru et al. [38] tested wounded L929 fibroblast cells with different concentrations of 25, 50, 75, and 100 mg/mL of biosynthesized *Blumea axillaris* ZnONPs. The wound healing was 95.37 ± 1.12% at 100 mg/mL concentration, after 24 h, while the control was 87.58 ± 1.06%.

Elattar et al. [32] cultured wounded BJ1 fibroblast cells with a melanin patch from *Aureobasidium pullulans* for 24 h. Cell migration was observed using phase-contrast microscopy at 0 and 24 h of incubation. It was found that BJ1 cells treated with melanin presented a migration ratio of 46.65% at 100 μg/mL and 42.71% at 250 μg/mL concentration, both at 24 h. Meanwhile, the control group at 24 h was 41.7%.

Mandarada et al. [37] tested different concentrations of M.FeONPs (*Arthrinium aureum* mycosynthesized iron-oxide nanoparticles) and FeONPs (iron-oxide nanoparticles) in wounded HUVEC cells and monitored for 0, 4, and 8 h. The findings suggested that the endothelial cell migration was increased significantly by M.FeONPs up to 8 h in a concentration-dependent manner compared to FeONP-treated and untreated cells. Also, the M.FeONP treatment at 10 μg/mL concentration showed a slightly enhanced wound closure compared with the positive control.

El-Sayed et al. [34] used human skin fibroblasts to evaluate the effects of selenium nanoparticles (SeNPs) synthesized by four endophytic fungi: *Aspergillus terreus*, *Aspergillus quadrilineatus*, *Aspergillus ochraceus*, and *Fusarium equiseti*. All four treatments demonstrated lower wound width compared to the control. *A. terreus* showed 1.63 ± 0.23%, *A. quadrilineatus* 1.63 ± 0.35%, *A. ochraceus* 1.75 ± 0.43%, and *F. equiseti* 1.78 ± 0.65%, while the control presented 2.08 ± 0.14%.

#### 3.6.2. In Vivo Studies

Among the reviewed studies, it was observed that 16 studies, representing 66%, included in vivo analyses, using different animal models and various methods for applying fungal extracts or molecules with healing potential. Seven of these studies were conducted with mice: Ali et al. [44] and Cueva-Clavijo et al. [43], which used BALB/c strain animals, Khan et al. [27] and Liu et al. [28], which used the T2DM strain, and Mandarada et al. [37], Mpenda et al. [48], and Shinta et al. [39]. Three articles used Sprague-Dawley rats: Aqilah et al. [42], Yenn et al. [41], and Wen et al. [29]; three used Wistar rats: Hamidi et al. [40], Eliwa et al. [35], and El-Bouseary et al. [33]; two used rabbits: Abdulmyanova et al. [49] and El-Zawawy et al. [36]; Salem et al. [31] used earthworms as an animal model.

All studies involved various types and sizes of wound incisions, as shown in the in vivo results (Table 3). Nine of the studies used non-infected wounds: Abdulmyanova et al. [49], Aqilah et al. [42], Cueva-Clavijo et al. [43], El-Zawawy et al. [36], Hamidi et al. [40], Liu et al. [28], Mandarada et al. [37], Mpenda et al. [48], and Salem et al. [31]. Two studies used wounds infected with MRSA, Ali et al. [44] and Yenn et al. [41], and the remaining studies involved wounds infected with different microorganisms. For applying the fungal extract to the wounds, different vehicles and concentrations were used, with no standardized protocol among the reviewed articles. However, four studies applied the extracts as ointments: Abdulmyanova et al. [49], Cueva-Clavijo et al. [43], El-Zawawy et al. [36], and Mandarada et al. [37].

All studies showed an increase in wound closure in the treated group of animals. The outcomes varied among the 16 studies. Ten studies evaluated wound healing, wound contraction, reduction in wound area, wound healing rate, and wound contraction rate, all expressed in percentage [28,29,33,35,37,40,42,43,48,49]. Five studies evaluated the diameter of the wound, wound and lesion size, expressed in mm or cm [27,31,36,41,44], and Shinta et al. [39] evaluated wound healing outcome through a collagen tissue density score.

Abdulmyanova et al. [49] reported that by day 4, the ulcer surface area had reduced by 27.4% compared to the initial size in the control group. By day 10, the wound area had decreased by 62.6%, and by day 16, the reduction reached 77.8% of the original size. In the experimental group, the wound area decreased by 28.0% on day 4, and by 72.3% on day 7. On day 10, the reduction became more significant, reaching 84.1% compared to the starting size. Epithelialization was fully completed by day 16, resulting in a smooth scar. Ali et al. [44] reported that the topical application of AP-AgNPs significantly reduced wound size on days 2 and 8 compared to infected, untreated control mice. By day 6, mice treated with AP-AgNPs or vancomycin exhibited notably improved wound healing. By day 8, the healing effect of AP-AgNPs was comparable to that of vancomycin, with both groups showing fully regenerated wound tissues.

Aqilah et al. [42] found that until day 14, the wound contraction in all treatment groups was significantly higher than that of the negative control, from 53.7 ± 22.4% in the negative control to 80.2 ± 2.0% in the CN extract group (extract from marine EF). There was a significant difference in the percentage of wound closure between the control and treatment groups throughout the observation. Treated groups with CN and SSD (silver sulphadiazine) presented 80.2–80.8%, whereas treated groups with MV (extract from marine EF) and ED (extract from marine EF) showed 77.5–72.5% healing when compared to the negative control (53.7%) on day 14. Cueva-Clavijo et al. [43] found that the wound had closed completely by 17 days after excision for three of the four treatments: Dc1, Dm2 maize, and Dm3. For the Dm4 maize, the wound was 0.04 mm^2^. After 72 h, the positive control wound area was 91 mm^2^; for the Dc1, Dm2 maize, and Dm4 maize, on average, it was 86 mm^2^, and the largest area was for Dc3 (90.45 mm^2^). The closure was 100% by day 17, except for Dm4 maize (99.9%).

According to El-Zawawy et al. [36], the average diameter of the rabbit skin burns was 12.2 ± 0.2 cm on the first day. Topical application of BA-based ointment in rabbits significantly increased the wound healing compared to the other groups. After 20 days of treatment, the wound area treated with BA reduced by about 2.0 cm more than that of SSD. Healing of wound areas did not differ significantly between groups I (BA) and II (SSD) on days 3 and 7, but differed significantly on days 10, 15, 20, 25, and 30. Total wound healing occurred 10 days sooner in the BA-treated group than in the SSD-treated group. Khan et al. [27] noted that the wound area reduction was higher in the Du-CD group as compared to that in the PBS group after day 3. Complete wound healing was observed on day 18 for the Du-CD group and on day 21 for the PBS group. Liu et al. [28] found that by day 7 after establishment, PC@MLIP MN significantly promoted diabetic wound healing speed, and its therapeutic effect was better than that of PC injection and the control, by accurately inhibiting local M1 macrophage activation.

El-Bouseary et al. [33] tested ANM extracts in wounds infected with *S. aureus*, in DMSO:Saline (1:1), and with concentrations of 50 mg/kg and 100 mg/kg. The negative control was the vehicle, and the positive control was gentamicin. This study reported the results in percentage of wound area, instead of percentage of wound closure, like the majority of studies. It was reported that, on day 2, the treated groups showed lower wound areas (31.82% and 12.77%) compared to the negative control group (90.84%), and similar to the positive control (23.77%). By day 4, all groups demonstrated a reduction in wound area, with the group that received the lowest treatment dose showing the best result (11.27%). On day 6, this same group maintained the lowest wound area (6.35%).

Eliwa et al. [35] tested RNM extracts (500 µg/mL and 1000 µg/mL) in non-infected wounds and wounds infected with Pseudomonas aeruginosa. Negative control was vehicle (saline 0.9%), and positive control was gentamicin. As reported by El-Bouseary et al. [33], this study also expressed the results in percentage of wound area, instead of percentage of wound closure. Uninfected wound areas of treated groups were 21.5% versus those of the negative control groups (35.8%). On day 6, the treated groups presented wound closure percentages of 28.3% and 20.2%, compared to the negative control group (47.9%). They also observed that treatment with 500 µg/mL of the endophytic fungus showed better results than treatment with 1000 µg/mL.

Hamidi et al. [40] tested EPS in non-infected wounds, at concentrations of 1, 5, and 10 mg/mL. Positive control was commercial phenytoin cream, and negative control was the vehicle (sterile distilled water). After 14 days, the highest percentage of wound closure (99.2%) was observed in the group treated with 10 mg/mL. This value was slightly higher than that of the positive control group (98.3%).

Mandarada et al. [37] showed that the mice treated with 1% M. FeONPs exhibited the most rapid wound healing, and wound healing capability was also observed in the group receiving 0.8% M.FeONPs. In 7 days, complete wound healing was achieved, compared to the FeONP-treated group and the untreated group. The rate of wound healing of M.FeONPs was concentration-dependent (1% ≥ 0.8% ˃ 0.5% ˃ 0.1%). The control group showed the slowest rate of wound healing. M.FeONP (1%, 0.8%, 0.5%, 0.1%) treatments showed better results, followed by 1% FeONPs, the vehicle control, and the untreated group. Mpenda et al. [48] presented that animals treated with FUCE 1 had the highest percentage of wound contraction on day 15 post-treatment and showed an increase in the closure compared with animals in the control group. Salem et al. [31] demonstrated that 15 mg of PsEAE with Vaseline showed significant and fast wound healing after 5 days. Groups with 5 mg of PsEAE with Vaseline and 10 mg of PsEAE with Vaseline showed increased wound healing after six days of treatment. However, the group that received only Vaseline exhibited an improvement in the wound healing process after 20 days.

Shinta et al. [39] evaluated tissue regeneration of different EF extract concentrations (5%, 10%, 15%) in rat skin wounds infected with *Candida albicans*. Wound-healing properties were assessed through histopathological analysis, conducted at 7 and 14 days post-treatment, and scored (from 0 to 4) according to collagen fiber density, in which 0 represented no collagen fibers and 5 a very tight density of collagen fibers. Controls with no treatment scored 0, while extracts at 5% scored 1 and 3, at 7 and 14 days post-treatment, respectively. Extracts at 10% and 15% exhibited minimal tissue improvement.

Yenn et al. [41] showed that the topical application of fraction Ma10 decreased the diameter of the wound compared to the placebo control. The wounds that received the drug control and 2% fraction Ma10 were fully recovered; the diameter of the wound was concentration-dependent, and higher concentration showed smaller wounds. Wen et al. [29] observed wound closure in the AgNP group after 7 days. The AgNP group also showed an improvement in the wound’s appearance. Wound closure rate (%) of the AgNP-treated group significantly exceeded that of the control group (on days 3, 7, 14, and 21) and was up to 95.54 ± 2.70% on day 21, suggesting an almost complete wound closure. These observations showed that wound healing was accelerated by AgNPs.

### 3.7. Risk of Bias

#### 3.7.1. In Vitro Studies

According to the scores for each in vitro study (Table 4), the overall quality of papers varied from moderate to high. Three of the nine studies achieved high quality [37,38,46]. Evaluating the fungus source and isolation criteria, all studies were qualified as high-quality, except for Elattar et al. [32], Hu et al. [45], and Naveen et al. [47], which received a quality of evidence of moderate. Besides presenting a detailed extraction method of the EF, Elattar et al. [32] and Hu et al. [45] did not mention the plant species host from the EF used in the work. Meanwhile, Naveen et al. [47] did not specify the geographic location of the plant species, nor the information from the collection, nor the part of the plant used in the extraction. Nehru et al. [38], despite mentioning the geographic location of the collection, did not mention the part of the plant used in the extraction. All studies described the fungal fermentation.

Regarding the extraction conditions, there was no study that received a quality of evidence lower than ‘moderate’. Araújo [26], El-Bouseary et al. [33], Eliwa et al. [35], El-Sayed et al. [34], Hu et al. [46], Mandarada et al. [37], Naveen et al. [47], and Nehru et al. [38] were classified as having high quality of evidence because of the description of the extraction methods, including the amount of biomass or supernatant used, the solvent, and the final product used in the assays. Farghali et al. [51] did not mention the proportion of solvent during extraction; Elattar et al. [32] did not report the vehicle of the molecule (melanin), nor did Hu et al. [45].

Since all studies provided full information about the wound-healing assay methodology (cell line under the purpose of the study, dose, timing, and use of controls), all works received high-quality ratings of evidence, except for El-Bouseary et al. [33], Eliwa et al. [35], and El-Sayed et al. [34], who did not mention the dose used in the assay. However, for the mechanism of action, only Mandarada et al. [37] received a high score, followed by Nehru et al. [38], with a moderate score. All the other studies received low-quality evidence scores in this criterion. Mandarada et al. [37] performed assays for the elucidation of the mechanism of action, signaling pathway, biochemical properties, biodistribution, and histopathology analysis. Through these methods, the authors concluded that the pro-angiogenic activity of M.FeONPs is linked to the upregulation of NOX (NADPH oxidase 2) and activation of the PI3K/Akt/MAPK pathway. Based on these results, M.FeONPs could be a promising bio-compatible and pro-angiogenic agent for treating various vascular-related diseases, including wound healing. Nehru et al. [38] analyzed the ZnONPs’ characteristics and stability. The authors could show evidence of some biological properties, such as antimicrobial activity, antioxidant, antidiabetic, anti-inflammatory, cytotoxic, and wound healing. The authors found that the synergistic interaction between ZnONPs and bioactive compounds produced by EF may contribute to their potent biological properties. Potential mechanisms of action were not discussed, nor were assays performed to explain them in other studies.

#### 3.7.2. In Vivo Studies

The selected in vivo studies (16) were evaluated according to the CAMARADES checklist (Table 5). The scores varied from 3 to 8, with Ali et al. [44] and Yenn et al. [41] scoring 3 points, while Liu et al. [28] scored 8 points. All studies were published in peer-reviewed journals, all of them presented the animal model, and none of them presented a sample size calculation. Liu et al. [28] were the only authors to state a blind wound induction and a blinded assessment of outcome. All studies avoided anesthetics with significant intrinsic neuroprotective activity, except for Ali et al. [44], El-Bouseary et al. [33], and Eliwa et al. [35], who treated in vivo models with ketamine and xylazine, which have neuroprotective activity when combined. Studies from Abdulmyanova et al. [49], Ali et al. [44], Salem et al. [31], and Yenn et al. [41] did not present any statement of compliance with regulatory requirements, and Abdulmyanova et al. [49], Aqilah et al. [42], and Yenn et al. [41] did not report a statement regarding possible conflict of interest. Ali et al. [44], Khan et al. [27], Mandarada et al. [37], Mpenda et al. [48], Shinta et al. [39], and Yenn et al. [41] did not report any statement of control of temperature.

## 4. Discussion

This systematic review evaluated the evidence that EFs are sources of biomolecules that promote wound healing. After a comprehensive search in the scientific literature, 24 works that tested extracts/biomolecules from EF in wound healing were found. From these, 16 performed in vivo assays, 11 conducted in vitro assays, and three performed both in vivo and in vitro assays. All studies presented correlations between EF extracts/biomolecules and wound-healing properties, and the tested group showed better wound-healing properties than the negative control group. Other biological properties were also evaluated throughout the studies, including antimicrobial, anti-inflammatory, antioxidant, cytotoxic, and antibiofilm activities, among others. These extracts were administered in wound-healing assays in forms such as nanoparticles, ointments, crude extracts, Vaseline, petroleum jelly, and saline solution. Doses varied immensely between studies, being expressed as percentage (%), μg/mL, μmol/mL, mg/mL, or mg, and some did not present this information.

From this systematic review, it is possible to note that most extraction methods included both solid–liquid extraction and liquid–liquid extraction, often with ethyl acetate as the solvent. Methanol, ethanol, water, ethanol precipitation, and dichloromethane were used in some studies. Because ethyl acetate is a more non-polar solvent, it is possible to infer that most of the molecules targeted in the study are non-polar. The few studies that included the EF extract main compound identified apolar ones: melanin, benzoic acid, and purpurolide (sesquiterpene). The studies that used precipitation with ethanol as the extraction method identified its main compound as polysaccharides, and two studies that used distilled water as the solvent used nanoparticles as the vehicle of the formulation. These results suggest that mainly non-polar compounds from EF extracts promote wound healing activity, which can be pigments, terpenoids, phenolic compounds, and flavonoids, the main non-polar compounds isolated from fungi. However, polar compounds from EF, such as exopolysaccharides (EPS), as reported by Hu et al. [45] and Hamidi et al. [40], have been reported for their biological activities, with potential application in nutraceutical and pharmaceutical industries [53].

Works were published from 2014 to 2026, showing how current this topic is. The oldest study was from Yenn et al. [41], who performed an in vivo assay with rat models. The most recent study found was by El-Sayed et al. [34], published in January 2026. No studies published between 10 March and the submission date were included. In the most recently published included study, the authors developed an optimized microbial process for nanomaterial production, with a focus on subsequent scale-up. Sodium selenite was added to the fungal culture to obtain selenium nanoparticles without the need for additional processing steps. The authors systematically evaluated cultivation parameters and the application of gamma irradiation to increase nanoparticle yield. The main metabolites presented in the nanoparticles were fatty acids and hydrocarbon compounds, which demonstrated wound healing and anticancer activities. These findings are significant, as they highlight the importance of investigating not only the bioactive properties of EF extracts but also process integration and optimization from an industrial perspective. In vitro studies demonstrated that treated cells exhibited faster wound closure compared to untreated cells, depending on the concentration and exposure time. This suggests that compounds derived from fungi can stimulate and accelerate the wound healing process. In line with this, in vivo studies also showed that treated groups achieved faster wound closure compared to both positive and negative control groups. Although these experiments are conducted over longer periods, due to the greater complexity of in vivo systems, the results are consistent with those observed in in vitro studies. Overall, the findings indicate that in vitro assays can serve as good predictors of the wound healing potential of these fungal compounds, while in vivo studies allow for a more comprehensive analysis of the healing process.

There was considerable variability in the positive controls used among the analyzed studies, including vancomycin [44], silver sulfadiazine [42], saline solution [36], Silverex [37], chloramphenicol [41], gentamicin [33,35], 1% phenytoin cream [40], Ulcoderma [43], and povidone-iodine [29]. In addition, the other six studies did not use positive controls. This heterogeneity directly affects the comparability of the results, as these compounds have different mechanisms of action. Consequently, the efficacy of the evaluated treatments may vary depending on the reference drug used. Thus, the lack of standardization of positive controls represents a limiting factor for direct comparisons between studies.

Analyzing the risk of bias of in vitro studies, Hu et al. [46], Mandarada et al. [37], and Nehru et al. [38] received high quality scores, while the other nine studies received moderate scores. In general, there was a good description (in the studies themselves or in previously cited studies of the authors) of all the upstream steps. However, Cueva-Clavijo et al. [43] and Elattar et al. [32] did not indicate the host plant, which can compromise the reliability of results. Cueva-Clavijo et al. [43] also did not report the fungus growth conditions (temperature, rotation, and time of cultivation), while Araújo [26], Farghali et al. [51], Liu et al. [28], Mandarada et al. [37], and Naveen et al. [47] did not report the agitation of cultures. Still in the upstream, Shinta did not report the extraction method. This data regarding the methodology of the studies is crucial for their reproducibility. Its absence compromises their reliability. In the upstream, most of the limitations were a lack of extract concentration used in vitro assays, and the molecule identification or mechanism of action evaluation.

In the CAMARADES checklist of the selected studies, it is important to note that 7 out of 16 in vivo studies scored ≤5 out of 10. Even though all studies were published in peer-reviewed journals, they lack critical information in their methodology. Nine studies made statements of temperature control and reported the animal model, and 10 included the randomization of treatment or control; only Liu et al. [26] reported blinded wound induction and blinded assessment of outcome; 12 avoided the use of anesthetics with significant intrinsic neuroprotective activity; only El-Bouseary et al. [33] and Eliwa et al. [35] made sample size calculations; 11 stated compliance with regulatory requirements; and 13 included a conflict of interest section. These results indicate a larger estimation of the tested groups’ outcomes and a high risk of bias, especially in the studies scoring ≤5. All in vivo studies have low n for each group (3–8), which by itself increases the risk of bias. Therefore, methodological rigor is required for trustworthy and more accurate outcomes.

The meta-analysis could not be carried out due to some factors, firstly the high heterogeneity of outcomes: WCR (%), wound area remaining (%), diameter (mm or cm), and histopathological analysis (collagen density score). Most of the in vivo studies presented the n used in each group, except for Khan et al. [27], who did not present the n; results were expressed with the average, and there was no indication of raw data. In vitro, studies used different extraction methods, cell lines, types, and extract concentrations. Some studies included positive controls, which varied, and others did not. In addition, some wounds in in vivo models were infected with different microorganisms, which would make for an inconsistent comparison amongst them. And, finally, different animal models were tested. These observations indicate a lack of standardization of in vivo wound-healing assays. For further pre-clinical and clinical trials, all of these criteria must be standardized with rigor, and cytotoxicity data must be included.

## 5. Conclusions

Through this systematic review, it is possible to conclude that EFs are potential sources of biomolecules with wound-healing properties, associated with properties such as antimicrobial, antioxidant, anti-inflammatory, antibiofilm, antiangiogenesis, and coagulant, which can promote benefits in burns, chronic diseases (i.e., diabetes mellitus, vascular disorders), and bleeding from a wound. However, results indicate a need for standardization of outcomes and experimental protocols, in both in vivo and in vitro studies. These findings further highlight the need for additional studies using standardized protocols, as well as research on the molecules involved in wound healing and their mechanisms of action.

## Figures and Tables

**Table 2:** Synthesis of assay conditions and results from in vitro studies.

Reference	Extract	Concentration	Cell Line	Time (h)	Results
De Araújo [26]	Raw extract	10 μg/mL	3T3	24	One extract promoter showed higher wound closure than the control.
Elattar et al. [32]	Melanin patch	100 mg/mL	BJ1	0–24	Wound closure > 45% at 24 h.
El-Bouseary et al. [33]	ANM	ND	W138	0, 24, 48	Wound closure of 99.68% after 48 h in the test group, and 83.37% in the control group.
El-Sayed et al. [34]	SeNPs	ND	HSF	0, 24, 48, 72	Wound area of treated groups decreased significantly in comparison to the positive control group.
Eliwa et al. [35]	ERM	ND	WI38	0, 24, 48	Wound closure of 94.66% after 48 h in the ERM-treated group, and 83.37% in the control group.
Farghali et al. [51]	EPR	ND	WI38	0, 24, 48	Wound closure > 65% in the test group and 13% in the control group after 24 h. Wound closure is higher than 99% after 48 h.
Hu et al. [46]	Tp-AgNPs	1, 5, 10 μg/mL	NIH3T3	0–38	Wound closure after 24 h.
Hu et al. [45]	TEPS1 and TEPS2	ND	HEK293	0–36	Wound closure of TPS1 at 36 h. Wound closure of TPS2 > control.
Mandarada et al. [37]	M.FeONPs	10 μg/mL	HUVEC	0, 4, 8	Slightly enhanced closure compared to the positive control.
Naveen et al. [47]	GA-CU-CeO_2_ NCs	125 μg/mL	NIH3T3	0–36	Increased proliferation and cell migration at 12 h, and wound closure improved in all tested groups after 24 h.
Nehru et al. [38]	*Blumea axillaris* ZnONPs	100 mg/mL	L929	24	Wound closure > 95% at 24 h.

3T3, WI38, NIH3T3, and L929 cell lines: fibroblasts; ANM: *Aspergillus niger* ethyl acetate extract; EPR: raw extracts of *Penicillium rubens*; ERM: ethyl acetate extract of *R. mucilaginosa*; GA-CU-CeO_2_ NCs: *Penicillium radiatolobatum* gum arabic–curcumin nanocomposites; HEK293: human embryonic kidney cell line; HSF: human skin fibroblast cell line; HUVEC: endothelium of human umbilical cord vein-type cell; M.FeONPs: *Arthrinium aureum* mycosynthesized iron-oxide nanoparticles; ND: non-declared; SeNPs: selenium nanoparticles; TEPS1 and TEPS2: *T. purpureogenus* extracellular polysaccharides; Tp-AgNPs: *Talaromyces purpureogenus* silver nanoparticles; ZnONPs: zinc oxide nanoparticles.

**Table 3:** Synthesis of selected in vivo studies, their methods and outcomes.

Reference	Model	n	Size of Incision	Wound Infection	Vehicle	Dose	Positive Control	Negative Control	Time (Days)	Main Outcomes
Abdulmyanova et al. [49]	Albino rabbits (both sexes)	5	Thermal burn UV irradiation	No	Ointment	5%	Kamistad^®^ gel	No treatment	Up to 19	Wound closure of 84.1% after 10 days
Ali et al. [44]	BALB/c mice (male)	6	2 cm^2^	MRSA	Ethanol in propylene glycol	100 μg/mL	Vancomycin	Vehicle; saline	2, 4, 6, 8	At day 8, the AP-AgNP group was comparable to vancomycin group
Aqilah et al. [42]	Sprague-Dawley rats (male)	6	Hot water burn	No	Tween 20^®^ 10%	10%	SSD	Vehicle	3, 7, 10, 14	Treated group showed 72.5% of wound closure at day 14
Cueva-Clavijo et al. [43]	BALB/C mice (both sexes)	6	10 cm^2^	No	Ointment	75 mg/mL	Ulcoderma	Vehicle	0, 1, 3, 7, 11, 14, 17	100% wound closure at day 17
El-Bouseary et al. [33]	Wistar albino rats (male)	3	10 mm diameter	*S. aureus*	DMSO:Saline 1:1	50 and 100 mg/kg	Gentamicin	Vehicle	0, 2, 4, 6	Treated group wound area reduced to 6.35% after 6 days
Eliwa et al. [35]	Wistar albino rats (male)	3	10 mm diameter	*Pseudomonas aeruginosa* (P22)	Saline 0.9%	500 and 1000 μg/mL	Gentamicin	Vehicle	0, 2, 4, 6	Treated group improved healing in both uninfected and infected wounds, relative to negative control group.
El-Zawawy et al. [36]	White rabbits	6	Burn injury	No	Ointment	150 μg/mL	SSD ointment	No treatment	3, 7, 10, 15, 20, 25, 30	Total wound closure after 10 days
Hamidi et al. [40]	Wistar rats (male)	6	1 cm diameter	No	Sterile distilled water	1, 5, and 10 mg/mL	Commercial phenytoin cream	Vehicle	3, 7, 10, 14	Wound closure in treated groups was dose-dependent, achieving 99.2%, which was comparable to positive control groups.
Khan et al. [27]	Balb/c mice	ND	10 mm diameter	*S. aureus*	CDs	2 mg/mL	ND	PBS	0, 7, 14, 21	Complete wound healing after 18 days
Liu et al. [28]	C57BL/6 mice (male)	6	1 cm diameter	No	Gelatin methacryloyl-based microneedles	10 μmol/L	ND	PBS	0, 7	By day 7, treated groups significantly promoted diabetic wound healing speed
Mandarada et al. [37]	C57BL/6 J mice (male)	5	6 mm diameter	No	FeONP Vaseline Ointment	1% (*w*/*w*)	Hematoxylin and eosin	FeONP Vaseline Ointment	0, 3, 5, 7	Complete wound healing was achieved in 7 days
Mpenda et al. [48]	Mice	5	1 cm diameter	No	DMSO 10%	30, 50, 70 μg/mL	ND	Vehicle	3, 6, 9, 12, 15	Treated group presented the highest percentage of wound healing in 15 days
Salem et al. [52]	Earthworms	5	ND	No	Vaseline	5, 10, 15 mg	ND	Vehicle	Up to 20	Treated groups showed increased wound healing after 6 days
Shinta et al. [39]	White rats (male)	6	2 cm in length and 2 mm depth	*C. albicans*	ND	5, 10, 15%	ND	No treatment	7, 14	Treated groups with extract at 5% showed high collagen fiber density
Yenn et al. [41]	Sprague-Dawley rats (both sexes)	8	10% of the body area	MRSA	100% of petroleum jelly	1, 2%	Chloramphenicol	Vehicle	1, 3, 7	The diameter of the wound in treated group was decreased compared to control
Wen et al. [17]	Sprague-Dawley rats	7	1.8 cm diameter	Mixture of *S. aureus*, *P. aeruginosa*, and *E. coli*	AgNPs	ND	Povidone-iodine	Saline solution	0, 3, 7, 14, 21	The treated group exhibited 95.5% of wound healing by day 21

AgNPs: silver nanoparticles; BA: benzoic acid; CDs: carbon dots; DMSO: dimethyl sulfoxide; FeONPs: iron-oxide nanoparticles; MRSA: methicillin-resistant *Staphylococcus aureus*; n: each group’s population; ND: no data; PBS: phosphate-buffered saline; SSD: sulphadiazine.

**Table 4:** Risk of bias in individual in vitro studies.

Reference	Fungal Source and Isolation	Extraction Conditions	Wound- Healing Assay	Mechanism of Action	Overall Quality ^1^
Araújo [26]	✓✓✓	✓✓✓	✓✓✓	✓	✓✓
Elattar et al. [32]	✓✓	✓✓	✓✓✓	✓	✓✓
El-Bouseary et al. [33]	✓✓✓	✓✓✓	✓✓	✓	✓✓
Eliwa et al. [35]	✓✓✓	✓✓✓	✓✓	✓	✓✓
El-Sayed et al. [34]	✓✓✓	✓✓✓	✓✓	✓	✓✓
Farghali et al. [51]	✓✓✓	✓✓	✓✓✓	✓	✓✓
Hu et al. [46]	✓✓✓	✓✓✓	✓✓✓	✓	✓✓✓
Hu et al. [45]	✓✓	✓✓	✓✓✓	✓	✓✓
Mandarada et al. [37]	✓✓✓	✓✓✓	✓✓✓	✓✓✓	✓✓✓
Naveen et al. [47]	✓✓	✓✓✓	✓✓✓	✓	✓✓
Nehru et al. [38]	✓✓	✓✓✓	✓✓✓	✓✓	✓✓✓

^1^ Overall Quality of Evidence: ✓ low; ✓✓ moderate; ✓✓✓ high.

**Table 5:** CAMARADES checklist of the included in vivo studies and their respective scores.

Reference	1	2	3	4	5	6	7	8	9	10	Total
Abdulmyanova et al. [49]	Y	Y	N	N	N	Y	Y	N	N	N	4
Ali et al. [44]	Y	N	N	N	N	N	Y	N	N	Y	3
Aqilah et al. [42]	Y	Y	Y	N	N	Y	Y	N	Y	N	6
Cueva-Clavijo et al. [43]	Y	Y	Y	N	N	Y	Y	N	Y	Y	7
El-Bouseary et al. [33]	Y	Y	Y	N	N	N	Y	Y	Y	Y	7
El-Zawawy et al. [36]	Y	Y	Y	N	N	Y	Y	N	Y	Y	7
Eliwa et al. [35]	Y	Y	Y	N	N	N	Y	Y	Y	Y	7
Hamidi et al. [40]	Y	N	Y	N	N	Y	Y	N	Y	Y	6
Khan et al. [27]	Y	N	N	N	N	Y	Y	N	Y	Y	5
Liu et al. [28]	Y	Y	N	Y	Y	Y	Y	N	Y	Y	8
Mandarada et al. [37]	Y	N	Y	N	N	Y	Y	N	Y	Y	6
Mpenda et al. [48]	Y	N	N	N	N	Y	Y	N	Y	Y	5
Salem et al. [31]	Y	Y	Y	N	N	Y	Y	N	N	Y	6
Shinta et al. [39]	Y	N	Y	N	N	Y	Y	N	Y	Y	6
Yenn et al. [41]	Y	N	N	N	N	Y	Y	N	N	N	3
Wen et al. [29]	Y	Y	Y	N	N	Y	Y	N	Y	Y	7

(1) Publication in peer-reviewed journal; (2) statement of control of temperature; (3) randomization of treatment or control; (4) blinded wound induction; (5) blinded assessment of outcome; (6) avoidance of anesthetics with significant intrinsic neuroprotective activity; (7) animal model; (8) sample size calculation; (9) statement of compliance with regulatory requirements; and (10) statement regarding possible conflict of interest. Y: Yes; N: No.

## Data Availability

All data generated or analyzed are included in this review.

## Supplementary Materials

The following supporting information can be downloaded at https://www.mdpi.com/article/10.3390/microorganisms14040918/s1: Figure S1. General process of included studies, from the isolation of endophytic fungi from plants to the cultivation of endophytic fungi, the extraction of bioactive compounds from endophytic fungi, the characterization of bioactive compounds, and the bioactive evaluation of the compounds. Table S1: Search strategies with appropriate keywords and MeSH terms; Table S2: Exclusion criteria; Table S3: Excluded studies and criteria reasons.

## Abbreviations

The following abbreviations are used in this manuscript:

AgNP	Silver nanoparticles
ANM	*Aspergillus niger* ethyl acetate extract
BA	Benzoic acid
CAMARADES	Collaborative Approach to Meta-Analysis and Review of Animal Data from Experimental Studies
EF	Endophytic fungi
EPR	Extracts of *Penicillium rubens*
EPS	Exopolysaccharides
ERM	Ethyl acetate extract of *R. mucilaginosa*
GA-CU-CeO_2_ NCs	Gum arabic–curcumin nanocomposites
LLE	Liquid–liquid extraction
M.FeONPs	Mycosynthesized iron-oxide nanoparticles
PD	Potato Dextrose
PDB	Potato Dextrose Broth
PRISMA	Preferred Reporting Items for Systematic Reviews and Meta-Analysis
SeNPs	Selenium nanoparticles
SLE	Solid–liquid extraction
TEPS1 and TEPS2	*Talaromyces purpureogenus* extracellular polysaccharides
Tp-AgNPs	*Talaromyces purpureogenus* silver nanoparticles
WCR	Wound Closure Rate
ZnONPs	Zinc oxide nanoparticles

## References

[1] Wilkinson H.N., Hardman M.J. (2020). Wound healing: Cellular mechanisms and pathological outcomes. Open Biol..

[2] Velnar T., Bailey T., Smrkolj V. (2009). The Wound Healing Process: An Overview of the Cellular and Molecular Mechanisms. J. Int. Med. Res..

[3] Arslan N.P., Orak T., Ozdemir A., Altun R., Esim N., Eroglu E., Karaagac S.I., Aktas C., Taskin M. (2024). Polysaccharides and Peptides with Wound Healing Activity from Bacteria and Fungi. J. Basic Microbiol..

[4] Chopra H., Mishra A.K., Baig A.A., Mohanta T.K., Mohanta Y.K., Baek K.-H., Chopra H., Mishra A.K., Baig A.A., Mohanta T.K. (2021). Narrative Review: Bioactive Potential of Various Mushrooms as the Treasure of Versatile Therapeutic Natural Product. J. Fungi.

[5] Comino-Sanz I.M., López-Franco M.D., Castro B., Pancorbo-Hidalgo P.L. (2021). The role of antioxidants on wound healing: A review of the current evidence. J. Clin. Med..

[6] Oliveira A., Simões S., Ascenso A., Reis C.P. (2022). Therapeutic advances in wound healing. J. Dermatol. Treat..

[7] Croitoru A.-M., Ficai D., Ficai A., Mihailescu N., Andronescu E., Turculet S.C. (2020). Nanostructured fibers containing natural or synthetic bioactive compounds in wound dressing applications. Materials.

[8] Barreto R.S., Albuquerque-Júnior R.L., Pereira-Filho R.N., Quintans J.S., Barreto A.S., DeSantana J.M., Santana-Filho V.J., Santos M.R., Bonjardim L.R., Araújo A.A. (2013). Evaluation of wound healing activity of atranorin, a lichen secondary metabolite, on rodents. Rev. Bras. Farmacogn..

[9] Napavichayanun S., Aramwit P. (2017). Effect of animal products and extracts on wound healing promotion in topical applications: A review. J. Biomater. Sci. Polym. Ed..

[10] Shedoeva A., Leavesley D., Upton Z., Fan C. (2019). Wound healing and the use of medicinal plants. Evid.-Based Complement. Altern. Med..

[11] Hillman P.F., Lee C., Nam S.-J. (2022). Microbial natural products with wound-healing properties. Processes.

[12] Hashem A.H., Attia M.S., Kandil E.K., Fawzi M.M., Abdelrahman A.S., Khader M.S., Khodaira M.A., Emam A.E., Goma M.A., Abdelaziz A.M. (2023). Bioactive compounds and biomedical applications of endophytic fungi: A recent review. Microb. Cell Factories.

[13] Gao Y., Xu Y., Dong Z., Guo Y., Luo J., Wang F., Yan L., Zou X. (2025). Endophytic Fungal Diversity and Its Interaction Mechanism with Medicinal Plants. Molecules.

[14] Wen J., Okyere S.K., Wang S., Wang J., Xie L., Ran Y., Hu Y., Wen J., Okyere S.K., Wang S. (2022). Endophytic Fungi: An Effective Alternative Source of Plant-Derived Bioactive Compounds for Pharmacological Studies. J. Fungi.

[15] Phongpaichit S., Rungjindamai N., Rukachaisirikul V., Sakayaroj J. (2006). Antimicrobial activity in cultures of endophytic fungi isolated from *Garcinia* species. FEMS Immunol. Med. Microbiol..

[16] Vasundhara M., Baranwal M., Kumar A., Vasundhara M., Baranwal M., Kumar A. (2016). *Fusarium tricinctum*, An Endophytic Fungus Exhibits Cell Growth Inhibition and Antioxidant Activity. Indian J. Microbiol..

[17] Li S.-J., Zhang X., Wang X.-H., Zhao C.-Q. (2018). Novel natural compounds from endophytic fungi with anticancer activity. Eur. J. Med. Chem..

[18] Calvo-Gomez O., Eshboev F., Mullaiarova K., Egamberdieva D. (2025). Endophytic Bioactive Compounds for Wound Healing: A Review of Biological Activities and Therapeutic Potential. Microorganisms.

[19] Page M.J., Moher D., Bossuyt P.M., Boutron I., Hoffmann T.C., Mulrow C.D., Shamseer L., Tetzlaff J.M., Akl E.A., Brennan S.E. (2021). PRISMA 2020 explanation and elaboration: Updated guidance and exemplars for reporting systematic reviews. BMJ.

[20] Guimarães M.B., Helbourn C.C.B., Gonçalves G.O., Gonçalves M.B.M., dos Reis P.E.D., Magalhães P.O.M. Endophytic Fungi as a Promising Source of Bioactive Compounds for Wound Healing: A Systematic Review and Meta-Analysis. PROSPERO 2025, CRD420251054403. https://www.crd.york.ac.uk/PROSPERO/view/CRD420251054403.

[21] Team T.E. (2013). EndNote; EndNote 2025.

[22] Greenhalgh T. (1997). How to read a paper: Papers that summarise other papers (systematic reviews and meta-analyses). BMJ.

[23] Wanderley M.C.d.A., Neto J.M.W.D., Filho J.L.d.L., Lima C.d.A., Teixeira J.A.C., Porto A.L.F. (2017). Collagenolytic enzymes produced by fungi: A systematic review. Braz. J. Microbiol..

[24] Higgins J.P.T., Thomas J., Chandler J., Cumpston M., Li T., Page M.J., Welch V.A.E. (2024). Cochrane Handbook for Systematic Reviews of Interventions Version 6.5 (Updated August 2024).

[25] Macleod M.R., O’Collins T., Howells D.W., Donnan G.A. (2004). Pooling of Animal Experimental Data Reveals Influence of Study Design and Publication Bias. Stroke.

[26] Araújo J.F.O.d. (2018). Atividade Antibacteriana, Citotóxica e Cicatrizante In Vitro de Fungos Endofíticos Isolados de Plantas Medicinais: *Mimosa Tenuiflora* (Willd.) Poir., *Poincianella Pyramidalis* Tul. e *Acrocomia Aculeata* (Jacq.) Lodd. Ex Mart. Doctoral Thesis.

[27] Khan B., Zhang J., Durrani S., Wang H., Nawaz A., Durrani F., Ye Y., Wu F.G., Lin F. (2024). Carbon-Dots-Mediated Improvement of Antimicrobial Activity of Natural Products. ACS Appl. Mater. Interfaces.

[28] Liu Y.T., Xia G.Y., Chen Y.Y., Xia H., Xu J.J., Guo L.J., Lin S., Liu Y. (2023). Purpurolide C-based microneedle promotes macrophage-mediated diabetic wound healing via inhibiting TLR4-MD2 dimerization and MYD88 phosphorylation. Acta Pharm. Sin. B.

[29] Wen L., Zeng P., Zhang L.P., Huang W.L., Wang H., Chen G. (2016). Symbiosis theory-directed green synthesis of silver nanoparticles and their application in infected wound healing. Int. J. Nanomed..

[30] Ma Y.F., Wu X., Xiu Z.H., Liu X., Huang B.Y., Hu L., Liu J., Zhou Z.Y., Tang X.D. (2018). Cytochalasin H isolated from mangrove-derived endophytic fungus induces apoptosis and inhibits migration in lung cancer cells. Oncol. Rep..

[31] Salem S.H., El-Maraghy S.S., Abdel-Mallek A.Y., Abdel-Rahman M.A., Hassanein E.H., Al-Bedak O.A., El-Aziz F.E.-Z.A.A. (2022). The antimicrobial, antibiofilm, and wound healing properties of ethyl acetate crude extract of an endophytic fungus *Paecilomyces* sp. (AUMC 15510) in earthworm model. Sci. Rep..

[32] Elattar K.M., Ghoniem A.A., Al-Askar A.A., Bhgat El-Gazzar U., El-Hersh M.S., Elsherbiny E.A., Eldadamony N.M., Saber W.I.A. (2024). Melanin Synthesized by the Endophytic *Aureobasidium pullulans* AKW: A Multifaceted Biomolecule with Antioxidant, Wound Healing, and Selective Anti-Cancer Activity. Curr. Top. Med. Chem..

[33] El-Bouseary M.M., Eliwa D., Farghali M.H., Ragab A.E. (2025). Investigating the potential antibacterial, anti-biofilm, wound healing and anti-inflammatory activity of the extract of *Aspergillus niger* endophyte isolated from cucumber leaves: In vitro and in vivo study. BMC Microbiol..

[34] El-Sayed E.-S.R., Younis N.A., Easa S.M., Hussein H.G., Hamdy A.A. (2026). Improving Selenium Nanoparticles Bioproduction by Gamma Irradiated Endophytic Fungi and Their Cytotoxic and Wound Healing Activities. Egypt. J. Chem..

[35] Eliwa D., El-Bouseary M.M., Farghali M.H., El-Masry T.A., Ragab A.E. (2025). Investigation of antibacterial and wound healing activities of the extract of *Rhodotorula mucilaginosa* endophyte isolated from cucumber leaves. Sci. Rep..

[36] El-Zawawy N.A., Ali S.S., Khalil M.A., Sun J.Z., Nouh H.S. (2022). Exploring the potential of benzoic acid derived from the endophytic fungus strain *Neurospora crassa* SSN01 as a promising antimicrobial agent in wound healing. Microbiol. Res..

[37] Mandarada A.P., Roy A., Misra S., Patra C.R., Mutheneni S.R. (2025). Investigation of Pro-angiogenic and Wound Healing Activity of Mycosynthesized Iron-Oxide Nanoparticles. BioNanoScience.

[38] Nehru L., Kandasamy G.D., Sekar V., Alshehri M.A., Panneerselvam C., Alasmari A., Kathirvel P. (2023). Green synthesis of ZnO-NPs using endophytic fungal extract of *Xylaria arbuscula* from *Blumea axillaris* and its biological applications. Artif. Cells Nanomed. Biotechnol..

[39] Shinta D.Y., Widyastuti W., Primal D., Sonata H., Saryono S. (2024). Treatment of Endophytic Fungal Extracts in *Candida albicans*-Infected Wounds with Collagen Regeneration. J. Angiother..

[40] Hamidi M., Okoro O.V., Ianiri G., Jafari H., Rashidi K., Ghasemi S., Castoria R., Palmieri D., Delattre C., Pierre G. (2023). Exopolysaccharide from the yeast *Papiliotrema terrestris* PT22AV for skin wound healing. J. Adv. Res..

[41] Yenn T.W., Rashid S., Nurhaida, Zakaria L., Ibrahim D. (2014). In Vivo Anti-Mrsa Activity of *Penicillium Minioluteum* ED24. J. Appl. Pharm..

[42] Aqilah H.M., Norhayati A.S., Siti A.A. (2018). Wound healing properties in Sprague-Dawley rats of marine endophytic fungi extracts. Malays. Appl. Biol..

[43] Cueva-Clavijo R.I., Téllez-Téllez M., Aguilar-Marcelino L., Wong-Villarreal A., Acosta-Urdapilleta M.D.L., Castañeda-Ramírez G.S., Montañez-Palma L.F., Hernández-Núñez E. (2024). Evaluation of Ointments with *Daldinia eschscholtzii* in Wound Healing in an In Vivo Model. J. Med. Food.

[44] Ali E.M., Rajendran P., Abdallah B.M. (2024). Mycosynthesis of silver nanoparticles from endophytic *Aspergillus parasiticus* and their antibacterial activity against methicillin-resistant *Staphylococcus aureus* in vitro and in vivo. Front. Microbiol..

[45] Hu X.W., Saravanakumar K., Park S., Han K.S., Wang M.H. (2023). Isolation, Characterization, Antioxidant, and Wound Healing Activities of Extracellular Polysaccharide from Endophytic Fungus *Talaromyces purpureogenus*. Appl. Biochem. Biotechnol..

[46] Hu X.W., Saravanakumar K., Jin T.Y., Wang M.H. (2019). Mycosynthesis, characterization, anticancer and antibacterial activity of silver nanoparticles from endophytic fungus *Talaromyces purpureogenus*. Int. J. Nanomed..

[47] Naveen K.V., Sathiyaseelan A., Kim K.M., Wang M.-H. (2024). Physicochemical characterization, antioxidant, anti-inflammatory, and wound healing potential of cytocompatible Gum Arabic-Curcumin-Cerium oxide Nanocomposites. J. Drug Deliv. Sci. Technol..

[48] Mpenda F.N., Madaha G., Jacob F. (2024). Wound healing and coagulant activity of crude extract metabolites from fungal endophytes. Int. J. Second. Metab..

[49] Abdulmyanova L.I., Burieva M., Gulyamova T., Mavlanov S. (2023). Evaluation of Some Healing Properties of Ointment Based on Endophytic Melanins *Cladosporium* sp.*—HT*207. Univers. Ximiq Biol..

[50] Ali S.K., Mohamed F.M., El-Ghorab A.H., Hamed E.A., Aboel-Ainin M.A., Abdelgawad M.A., El-Adl K., Mohamed H.S. (2024). Biological activity and chemical characteristics studies of new oligomannose produced by *Erwinia gerundensis*. Carbohydr. Polym. Technol. Appl..

[51] Farghali M., Eliwa D., El-Bouseary M.M. (2025). Potential Antibacterial, Wound Healing and Anti-inflammatory Activities of *Penicillium rubens*, an Endophytic Fungus Isolated from the Leaves of *Cucumis sativus* L. Front. Sci. Res. Technol..

[52] Salem M.A., Mohamed O.G., Mosalam E.M., Elberri A.I., Abdel-Bar H.M., Hassan M., Al-Karmalawy A.A., Tripathi A., Ezzat S.M., Abo Mansour H.E. (2023). Investigation of the phytochemical composition, antioxidant, antibacterial, anti-osteoarthritis, and wound healing activities of selected vegetable waste. Sci. Rep..

[53] Liu J., Wang X., Pu H., Liu S., Kan J., Jin C. (2017). Recent advances in endophytic exopolysaccharides: Production, structural characterization, physiological role and biological activity. Carbohydr. Polym..

