# Supplementary material for: Endophytic Fungi as a Promising Source of Bioactive Compounds for Wound Healing: A Systematic Review
Source: Microorganisms. 2026 Apr 18;14(4):918. doi: 10.3390/microorganisms14040918 (PMC13118587; doi:10.3390/microorganisms14040918)
Supplement: Supplementary file 1 [file microorganisms-14-00918-s001.zip › microorganisms-4246083-supplementary.pdf]

Table S1. Search strategies with appropriated key words and MeSH terms.

**Database**	**Search Strategies**
PMC (March 10th, 2026)	((endophytic[All Fields] AND ("fungi"[MeSH Terms] OR "fungi"[All Fields] OR "fungus"[All Fields])) OR (endophytic[All Fields] AND ("microbiology"[Subheading] OR "microbiology"[All Fields] OR "fungi"[All Fields] OR "fungi"[MeSH Terms])) OR mycoendophyte[All Fields])) AND (wound healing[MeSH Terms])
PubMed (March 10th, 2026)	(endophytic fungus OR endophytic fungi OR mycoendophyte) AND (wound healing)
Scopus (March 10th, 2026)	(TITLE-ABS-KEY (endophytic AND fungus) OR TITLE-ABS-KEY (endophytic AND fungus) OR TITLE-ABS-KEY (mycoendophyte) AND TITLE-ABS-KEY (wound AND healing))
Science Direct (March 10th, 2026)	(endophytic fungus OR endophytic fungi OR mycoendophyte) AND (wound healing)
Web of Science (March 10th, 2026)	(((TS=(endophytic fungi)) OR TS=(endophytic fungus)) OR TS=(mycoendophyte)) AND TS=(wound healing)
Embase (March 10th, 2026)	('endophytic fungus'/exp OR 'endophytic fungus' OR 'endophytic fungus':ti,ab,kw OR mycoendophyte:ti,ab,kw) AND 'wound healing':ti,ab,kw

Google Scholar (March 10th, 2026)	allintitle: "endophytic fungus" OR "endophytic fungi" OR mycoendophyte "wound healing"
ProQuest™ Dissertation & Theses Citation Index (March 10th, 2026)	("endophytic fungi" OR "endophytic fungus" OR "mycoendophyte") AND "wound healing"

Table S2. Exclusion criteria.

Number	Exclusion criteria
1	Observational studies, reviews, conference abstracts, editorials and expert opinion
2	Inducted wounds in humans
3	Inducted wound in plant tissue
4	Cells and/or animals without inducted wounds
5	Extract and/or compounds from filamentous fungi, bacteria, endo-phytes, and any other organism rather than endophytic fungi
6	Compounds from non-identified endophytic fungi species
7	Synthetized molecules
8	Wound healing assays in cancer cells
9	Fungi isolated from other organisms rather than plants
10	Full text not found

Table S3. Excluded studies with reasons (n=20).

Study	Exclusion criteria
Ameen et al. (2021), Isolation, identification and bioactivity analysis of an endophytic fungus isolated from *Aloe vera* collected from Asir desert, Saudi Arabia (Ameen *et al.*, 2021)	8
Anwer et al. (2019), Wound healing process induced by a secondary metabolite produced by an endophytic fungus isolated from Caatinga (Anwer *et al.*, 2019)	1
Asiri et al. (2015), Penicillivinacine, antimigratory diketopiperazine alkaloid from the marine-derived fungus *Penicillium vinaceum* (Asiri, Badr & Youssef, 2015)	8
Britt et al. (2020), Differentially expressed genes in cotyledon of ewes fed mycotoxins (Britt, Noorai & Duckett, 2020)	8
Cao et al. (2022), A target and efficient synthetic strategy for structural and bioactivity optimization of a fungal natural product (Cao *et al.*, 2022)	8
El-Hady et al. (2024), Bioprocessing of camptothecin from *Alternaria brassicicola*, an endophyte of *Catharanthus roseus*, with a strong antiproliferative activity and inhibition to Topoisomerases (El-Hady *et al.*, 2024)	8
El-Sayed et al. (2024), Camptothecin bioprocessing from *Aspergillus terreus*, an endophyte of *Catharanthus roseus*: antiproliferative activity, topoisomerase inhibition and cell cycle analysis (El-Sayed *et al.*, 2024)	8
Eldeghidy et al. (2023), Production, bioprocessing and antiproliferative activity of camptothecin from *Aspergillus terreus*, endophyte of *Cinnamomum*	8

camphora: restoring their biosynthesis by indigenous microbiome of C (Eldeghidy *et al.*, 2023)	
Feng et al. (2016), A novel small molecule compound diaporine inhibits breast cancer cell proliferation via promoting ROS generation (Feng *et al.*, 2016)	8
Gao et at. (2023), Novel chlorinated and nitrogenated azaphilones with cyto-toxic activities from the marine algal-derived fungus *Chaetomium globosum* 2020HZ23 (Gao *et al.*, 2023)	8
Hu et al. (2025), Endophytic fungus *Umbelopsis* sp. TM01 as high-activity alternative to *Tricholoma matsutake* (Hu *et al.*, 2025)	9
Hulikere et al. (2016), Antiangiogenic, wound healing and antioxidant activity of *Cladosporium cladosporioides* (Endophytic Fungus) isolated from seaweed (*Sargassum wightii*) (Hulikere *et al.*, 2016)	8
Lu et al. (2022), Cytochalasin Q exerts anti-melanoma effect by inhibiting creatine kinase B (Lu *et al.*, 2022)	8
Qin et al. (2023), Secondary metabolites from *Pseudallescheria boydii* and their anti-melanoma activity (Qin *et al.*, 2023)	8
Rajulu et al. (2011), Chitinolytic enzymes from endophytic fungi (Rajulu *et al.*, 2011)	8
Refaat et al. (2024), Production and bioprocessing of epothilone B from *Aspergillus niger*, an endophyte of *Latania loddegesii*, with a conceivable biosynthetic stability: anticancer, anti-wound healing activities and cell cycle analysis (Refaat *et al.*, 2024)	8
Tang et al. (2017), Polyketides from the endophytic fungus *Phomopsis* sp. sh917 by using the one strain/many compounds strategy (Tang *et al.*, 2017)	8

Vasarri et al. (2022), Dihydroauroglaucin Isolated from the Mediterranean Sponge *Grantia compressa* Endophyte Marine Fungus *Eurotium chevalieri* Inhibits Migration of Human Neuroblastoma Cells (Vasarri *et al.*, 2022)	9
Yang et al. (2024), 12-O-deacetyl-phomoxanthone A inhibits ovarian tumor growth and metastasis by downregulating PDK4 (Yang *et al.*, 2024)	8
Zhan et al. (2007), Search for Cell Motility and Angiogenesis Inhibitors with Potential Anticancer Activity: Beauvericin and Other Constituents of Two Endophytic Strains of *Fusarium oxysporum* (Zhan *et al.*, 2007)	8

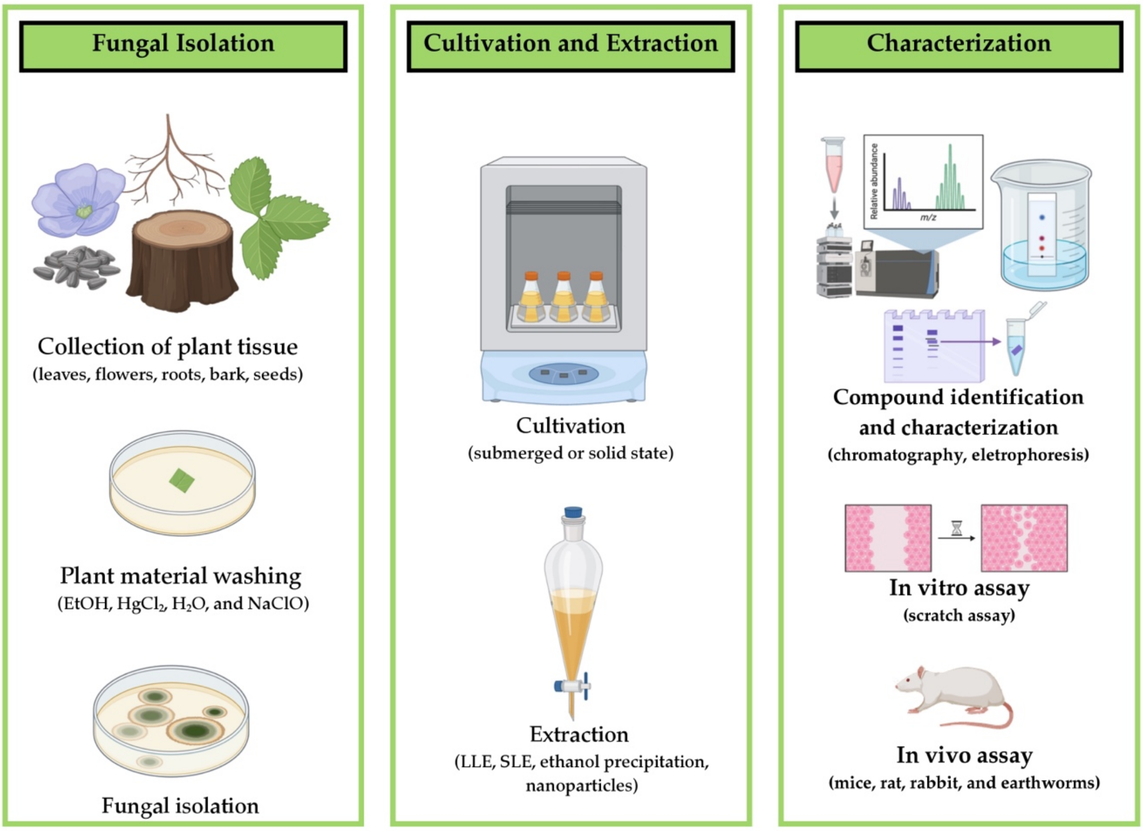

Figure S1. General process of included studies, from the isolation of endophytic fungi from plants to the cultivation of endophytic fungi, the extraction of bioactive compounds from endophytic fungi, the characterization of bioactive compounds, and the bio-active evaluation of the compounds.

References

AMEEN, F., STEPHENSON, S. L., ALNADHARI, S. & YASSIN, M. A. (2021). Isolation, identification and bioactivity analysis of an endophytic fungus isolated from *Aloe vera* collected from Asir desert, Saudi Arabia. *Bioprocess Biosyst Eng* **44**(6), 1063-1070.

ANWER, U. B., MINARINI, P., MARIA, D. A. & SOUZA, A. O. (2019). Wound healing process induced by a secondary metabolite produced by an endophytic fungus isolated from Caatinga.

ASIRI, I. A. M., BADR, J. M. & YOUSSEF, D. T. A. (2015). Penicillivinacine, antimigratory diketopiperazine alkaloid from the marine-derived fungus *Penicillium vinaceum*. *Phytochemistry Letters* **13**, 53-58.

BRITT, J. L., NOORAI, R. E. & DUCKETT, S. K. (2020). Differentially expressed genes in cotyledon of ewes fed mycotoxins. *Bmc Genomics* **21**(1), 680.

CAO, F., ZHANG, M. K., YANG, X., XU, C. X., CHENG, J. T., ZHAO, Q. W., WU, R., SHENG, R. & MAO, X. M. (2022). A target and efficient synthetic strategy for structural and bioactivity optimization of a fungal natural product. *European Journal of Medicinal Chemistry* **229**.

EL-HADY, N., ELSAYED, A. I., WADAN, K. M., EL-SAADANY, S. S. & EL-SAYED, A. S. A. (2024). Bioprocessing of camptothecin from *Alternaria brassicicola*, an endophyte of *Catharanthus roseus*, with a strong antiproliferative activity and inhibition to Topoisomerases. *Microb Cell Fact* **23**(1), 214.

EL-SAYED, A. S. A., ELSAYED, A. I., WADAN, K. M., EL-SAADANY, S. S. & ABD EL-HADY, N. A. A. (2024). Camptothecin bioprocessing from *Aspergillus terreus*, an endophyte of *Catharanthus roseus*: antiproliferative activity, topoisomerase inhibition and cell cycle analysis. *Microb Cell Fact* **23**(1), 15.

ELDEGHIDY, A., ABDEL-FATTAH, G., EL-SAYED, A. S. A. & ABDEL-FATTAH, G. G. (2023). Production, bioprocessing and antiproliferative activity of camptothecin from *Aspergillus terreus*, endophyte of *Cinnamomum camphora*: restoring their biosynthesis by indigenous microbiome of C. camphora. *Microb Cell Fact* **22**(1), 143.

FENG, X., YU, W., ZHOU, F., CHEN, J. & SHEN, P. (2016). A novel small molecule compound diaporine inhibits breast cancer cell proliferation via promoting ROS generation. *Biomedicine & Pharmacotherapy* **83**, 1038-1047.

GAO, Z. J., CAO, L. L., REN, H. P., YU, H. & WANG, Y. (2023). Novel chlorinated and nitrogenated azaphilones with cytotoxic activities from the marine algal-derived fungus *Chaetomium globosum* 2020HZ23. *Frontiers in Microbiology* **14**.

HU, L., DAI, K., GONG, C. H., HUANG, C. J., JIAO, S. & ZHANG, J. H. (2025). Endophytic fungus *Umbelopsis* sp. TM01 as high-activity alternative to *Tricholoma matsutake*. *Bioresource Technology* **422**.

HULIKERE, M. M., JOSHI, C. G., ANANDA, D., POYYA, J. & NIVYA, T. (2016). Antiangiogenic, wound healing and antioxidant activity of *Cladosporium cladosporioides* (Endophytic Fungus) isolated from seaweed (*Sargassum wightii*). *Mycology* **7**(4), 203-211.

LU, Y., ZHANG, P., CHEN, H., TONG, Q., WANG, J., LI, Q., TIAN, C., YANG, J., LI, S., ZHANG, Z., YUAN, H. & XIANG, M. (2022). Cytochalasin Q exerts anti-melanoma effect by inhibiting creatine kinase B. *Toxicology and Applied Pharmacology* **441**, 115971.

QIN, S.-L., DING, J.-X., HUANG, C.-Y., LI, J., YAO, M., LIU, Q.-P., WANG, W.-J. & YANG, X.-L. (2023). Secondary metabolites from *Pseudallescheria boydii* and their anti-melanoma activity. *Tetrahedron* **147**, 133663.

RAJULU, M. B. G., THIRUNAVUKKARASU, N., SURYANARAYANAN, T. S., RAVISHANKAR, J. P., EL GUEDDARI, N. E. & MOERSCHBACHER, B. M. (2011). Chitinolytic enzymes from endophytic fungi. *Fungal Diversity* **47**(1), 43-53.

REFAAT, S., FIKRY, E., TAWFEEK, N., EL-SAYED, A. S. A., EL-DOMIATY, M. M. & EL-SHAFAE, A. M. (2024). Production and bioprocessing of epothilone B from *AspergIllus niger*, an endophyte of *Latania loddegesii*, with a conceivable biosynthetic stability: anticancer, anti-wound healing activities and cell cycle analysis. *Microb Cell Fact* **23**(1), 229.

TANG, J.-W., WANG, W.-G., LI, A., YAN, B.-C., CHEN, R., LI, X.-N., DU, X., SUN, H.-D. & PU, J.-X. (2017). Polyketides from the endophytic fungus *Phomopsis* sp. sh917 by using the one strain/many compounds strategy. *Tetrahedron* **73**(26), 3577-3584.

VASARRI, M., VITALE, G. A., VARESE, G. C., BARLETTA, E., D'AURIA, M. V., DE PASCALE, D. & DEGL'INNOCENTI, D. (2022). Dihydroauroglaucin Isolated from the Mediterranean Sponge *Grantia compressa* Endophyte Marine Fungus *Eurotium chevalieri* Inhibits Migration of Human Neuroblastoma Cells. *Pharmaceutics* **14**(3).

YANG, C. X., XING, S. P., WEI, X., LU, J. F., ZHAO, G. S., MA, X. L., DAI, Z. T., LIANG, X., HUANG, W., LIU, Y. Y., JIANG, X. & ZHU, D. (2024). 12-O-deacetyl-phomoxanthone A inhibits ovarian tumor growth and metastasis by downregulating PDK4. *Biomedicine & Pharmacotherapy* **175**.

ZHAN, J., BURNS, A. M., LIU, M. X., FAETH, S. H. & GUNATILAKA, A. A. L. (2007). Search for Cell Motility and Angiogenesis Inhibitors with Potential Anticancer Activity:

Beauvericin and Other Constituents of Two Endophytic Strains of *Fusarium oxysporum*. *J Nat Prod* **70**(2), 227-32.
